# The feasibility and test–retest reliability of the Dutch Swal-Qol adapted interview version for dysphagic patients with communicative and/or cognitive problems

**DOI:** 10.1007/s11136-012-0202-y

**Published:** 2012-05-30

**Authors:** Jessie Lemmens, Gerrie J. J. W. Bours, Martien Limburg, Anna J. H. M. Beurskens

**Affiliations:** Zuyd University of Applied Sciences, Heerlen, The Netherlands

**Keywords:** Test–retest reliability, Feasibility, Dysphagia, Quality of life, Swal-Qol

## Abstract

**Purpose:**

The adaptation of the Dutch Swal-Qol questionnaire to an interview format suitable for dysphagic patients with communicative and/or cognitive problems and evaluation of the feasibility and test–retest reliability.

**Methods:**

An observational study with two measurements within a 2-week time period in a sample of 57 stroke patients with dysphagia in a nursing home environment. The interview version of the Swal-Qol was evaluated in the total group and in subgroups of patients with and without communicative and/or cognitive problems.

**Results:**

The constructed interview version was considered feasible from an expert’s and patient’s point of view. The overall score and seven subscales of the Swal-Qol showed an excellent test–retest reliability (*k* > 0.75), and two subscales were considered good (*k* > 0.60).

**Conclusions:**

This study showed that using a structured, and at the same time flexible, interview format tailored to the individual needs of stroke patients enhances the feasibility and does not compromise the test–retest reliability.

## Introduction

Health Related Quality of Life (HRQOL) scales are patient-reported outcome measures for gaining information about a patient’s own health situation [1, 2]. When assessing patients with communicative and/or cognitive problems, caution is needed when using traditional HRQOL scales since difficulties in understanding or answering may occur [3–5]. Several studies give recommendations for adjustments for patient groups with communicative and cognitive problems [6–9]. McHorney et al. [10] developed the Swal-Qol questionnaire to evaluate the impact of dysphagia on quality of life and was shown to be a reliable and valid tool for measuring quality of life in outpatients [11–14]. However, the current self-report version is not feasible for patients as half of them need assistance in filling it out [11, 13, 15]. Therefore, the aim of this study was to adapt the Swal-Qol questionnaire for dysphagic stroke patients with and without communicative and/or cognitive problems and evaluate its feasibility and test–retest reliability.

## Methods

### Design

A cross-sectional, clinimetric study design was used with two measurements in a 2-week time period in a sample of dysphagic stroke patients in a nursing home environment.

### Materials and methods

The Swal-Qol consists of 44 items divided in 10 scales regarding quality of life: (1) burden, (2) eating duration, (3) eating desire, (4) food selection, (5) communication, (6) fear, (7) mental health, (8) social functioning, (9) fatigue and (10) sleep that could be rated on a 5-point Likert scale. In addition, there are scales on symptom frequency, nutrition intake (tube, consistency of food and/or liquids), assistance with filling out, general health and some demographics [14]. We adapted the Dutch translation of the Swal-Qol [15] into an interview version by using a two-step response method with supportive visual aids [6–9]. The adaptation process is described in Box 1.

**Box 1:**
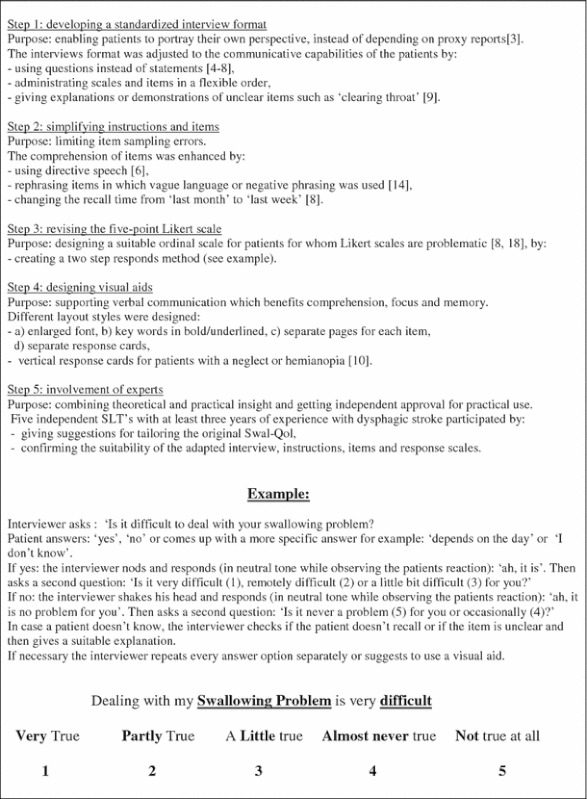
Swal-Qol adaptations, from self-report to interview version

### Participants

Stroke patients with dysphagia were recruited by speech and language therapists (SLTs) responsible for dysphagia treatment in nursing homes. Patients were eligible when they were physically and mentally fit enough to participate. Patients were excluded if they did not speak Dutch. The SLTs gave eligible patients oral and written information and asked permission for the interviewers to approach them. If patients were not able to give written consent, permission was asked in the presence of family members. If at 2-week follow-up patients did not recall giving consent, it was asked for again.

### Measurements

#### Patient characteristics

Communicative problems as dysarthria and aphasia were diagnosed by a SLT. Cognitive problems were defined as neuropsychological symptoms following stroke and were diagnosed by a psychologist. The severity of dysphagia was determined by feeding status. Dysphagia was considered ‘severe’ when patients were fed by tube (with and without oral intake), ‘moderate’ when there was oral intake with adjusted consistency and ‘mild’ in the case of normal oral intake with adaptive strategies.

#### Feasibility

Feasibility was defined as time to complete the interview, use of visual aids, item comprehension and burden from an observer’s and patient’s perspective. Burden was described as to what extent the interview had been tiresome for the patient. Regarding the patient’s perspective, two questions on a three-point scale about comprehension and burden were asked.

#### Test–retest reliability

Test–retest reliability was assessed by administrating two identical Swal-Qol interviews with the same interviewer. A 2-week time interval was considered enough time for patients not to remember their previous answers.

### Procedures

The SLTs and psychologist diagnosis were derived from the patients’ medical records to gather information on communication and cognition. Two trained assessors, one interviewer and one observer, conducted all the interviews. All patients were asked whether they preferred to use visual aids. The interviewer administered the Swal-Qol interview and wrote down the answers given by the patient; the observer reported the feasibility aspects. The use of visual aids and explanations were standardized in the second interview to guarantee that both interviews were identical. Before the second interview, the assessors checked whether no major changes had occurred in health status.

### Data analyses

The Predictive Analytics Software (PASW, version 18) program was used for data analyses. Patient characteristics were reported in frequencies. Subgroups were formed based on the presence of communicative and/or cognitive problems. Feasibility aspects were analyzed using descriptive techniques (i.e., mean, standard deviation, frequencies). Subscales with at least one incomplete answer were excluded from test–retest analyses. The test–retest reliability for each subscale and overall score was reported by the weighted kappa and is considered good between 0.40 and 0.75 and excellent above 0.75 [16, 17]. To compare the data with previous published studies, the Spearman’s rho correlation coefficients and the intraclass correlation coefficients (ICC) were calculated.

## Results

### Participants

A total of 61 patients met the inclusion criteria of whom 57 gave their consent and 56 participated in both interviews. Data were collected from April 2008–December 2009 in seven nursing homes in the Netherlands. The average age was 75.1 (±SD 12.1). Communication problems were present in 30 (53 %) patients. The most frequent diagnosis was aphasia or dysarthria (*n* = 24). Three patients had both speech and language problems. Cognitive problems occurred in 12 (21 %) patients and existed mostly of amnesia or neglect (*n* = 11; Table 1). Two patients had cognitive as well as communicative problems of whom one only participated in the feasibility study.

**Table 1:** Sample characteristics (*n* = 57)

	*n* (%)
Gender	
Male	24 (42)
Age	
Mean (SD)	75.1 (12.1)
Min	46
Max	94
Highest completed education	
Elementary school	22 (39)
High school	16 (28)
Vocational training	14 (25)
University	5 (9)
Dysphagia	
Severe: tube feeding	9 (16)
Moderate: oral intake with adjusted consistency	25 (47)
Mild: normal oral intake with adaptive strategies	21 (37)
Communication and/or cognitive problems^a^	41 (72)
Communication problems^a^	33 (58)
Aphasia (language)	12 (21)
Dysarthria (speech/voice)	12 (21)
Buccofacial/verbal apraxia (speech)	5 (9)
Unclassified diagnosis (speech/language)	4 (7)
Cognitive problems (neuropsychological symptoms)^a^	12 (21)
Amnesia	6 (11)
Neglect/hemianopia	6 (11)
None	16 (28)

^a^Patients can experience several problems in communication and/or cognition

### Feasibility

The average time to complete the interviews was 41 (±SD 28) min, and 29 (37 %) patients needed 30 min or more. There were no major differences between the group with and without communicative and/or cognitive problems.

Visual aids were used in 30 (53 %) interviews. Half of the patients without communicative or cognitive problems (*n* = 8) preferred visual aids. Only the enlarged font questionnaire (*n* = 11: 19 %) and separate response cards (*n* = 18: 32 %) were applied, mainly to help patients sustain attention.

Difficulties in comprehension were reported in all 44 items of the Swal-Qol. In 12 (21 %) interviews, a pause was needed, prolonging the administration time. Item comprehension was easy to 45 (78 %) patients and difficult to three (5 %) patients. The majority of the patients (82 %) found the burden acceptable to low.

### Test–retest reliability

The average time between the two interviews was 14.9 (± SD 3.1) days with a minimum of 7 and maximum of 24 (range = 17) days. Due to missing data, the sample size per subscale varied between 48 and 56 patients (Table 2). The weighted kappa was excellent (*k* > 0.75) for the overall score and seven subscales of the total group (*n* = 56), the group with (*n* = 40) and without communicative and/or cognitive problems (*n* = 16). It was good for subscale ‘fear’ (*k* = 0.675/0.677/0.660) and ‘fatigue’ (*k* = 0.713/0.736/0.631). A further comparison between communicative and cognitive problems showed that all scales were excellent for the group with communicative problems (*n* = 30). The sample size of the cognitive impaired group was considered too small for analysis. There were no major differences in test–retest reliability between the use of the weighted kappa, the Spearman’s rho and ICC (Table 2).

**Table 2:** Test–retest reliability of the Swal-Qol subscales (*n* = 56)

Subscale (# items)	*n*	Total group (weighed kappa)	Total group (Spearman’s rho/ICC)	Subgroups (weighed kappa)	
				Problems with communication and/or cognition (*n* = 40)	No problems with communication/cognition (*n* = 16)
1 Burden (2)	56	0.849	0.854/0.850	0.862	0.815
2 Eating duration and desire (5)	54	0.822	0.828/0.817	0.811^a^	0.856
3 Dysphagia symptoms (14)	54	0.940	0.934/0.941	0.960^a^	0.779
4 Food selection (2)	53	0.823	0.834/0.818	0.836^a^	0.804^a^
5 Communication (2)	56	0.786	0.777/0.789	0.790	0.761
6 Fear (4)	53	0.675	0.715/0.678	0.677^a^	0.660^a^
7 Mental health (5)	55	0.898	0.877/0.891	0.879	0.946^a^
8 Social functioning (5)	48	0.908	0.909/0.909	0.931^a^	0.785^a^
9 Fatigue and sleep (5)	55	0.713	0.710/0.714	0.736	0.631^a^
Overall score (44)	48	0.953	0.951/0.952	0.960^a^	0.924^a^

^a^ *n* is smaller than the group size given at the top of each subgroup

## Discussion

This study showed that using a structured, and at the same time, flexible interview format tailored to the individual needs of patients enhances the feasibility and does not compromise the reliability for dysphagic stroke patients with problems in communication and cognition.

In general, an interview format is more time-consuming and requires more resources and training, but we demonstrated that our adaptations led to a scale applicable within 30 min in half of the cases. In comparison with previous Swal-Qol studies, with an average fill-out time between 14 and 30 min [11, 13, 15], time to complete our interviews was much longer, mostly due to storytelling. However, the length of the Swal-Qol interview is considered feasible, since the majority of patients did not experience a heavy burden and were satisfied with the time to complete and the attention given by the interviewer.

Caution is needed when comparing our test–retest reliability results with previous studies, since these studies used the self-report version and excluded patients with communicative and cognitive problems. Their population was not limited to stroke, the sample sizes were small (*n* < 40), and most patients were not living in nursing homes. Despite these differences, the results of our test–retest reliability analysis are largely comparable with previous studies.

The absence of recent objective test results to estimate the communicative and/or cognitive problems might have influenced the group composition for the subgroup analysis. Since our population consisted of a group of very frail elderly, it was not considered ethical or practical to use additional standardized tests; instead, the diagnosis was derived from medical records. As group comparisons showed no major differences between feasibility aspects and test–retest reliability, it is not likely that this had a large impact. Moreover, by allowing a group of patients to participate who are usually excluded from research, we believe our data are based upon a more representative group of dysphagic stroke patients than reported thus far.

By tailoring measurement instruments for specific patient groups without compromising the clinimetric properties, data on subjective health status can be collected in a larger and more representative population, which benefits both research and clinical purposes. We think that more specific instruments in different domains can be adapted for use in this specific group of patients.

## References

[1] Bullinger M, Anderson R, Cella D, Aaronson NK (1993). Developing and evaluating cross cultural instruments: from minimum requirements to optimal models. Quality of Life Research.

[2] Williams L-S (1999). Measuring quality of life in a way that is meaningful to stroke patients. Neurology.

[3] Price CIM, Curless RH, Rodgers H (1999). Can stroke patients use visual analogue scales. Stroke.

[4] Williams L-S (1999). Development of a stroke-specific quality of life scale. Stroke.

[5] Hilari K (2003). Stroke and aphasia quality of life scale-39 (SAQOL-39), evaluation of acceptability, reliability, and validity. Stroke.

[6] Doesborgh S (2003). Linguistic deficits in the acute phase of stroke. Journal of Neurology.

[7] Lloyd V, Gatherer A, Kalsy S (2006). Conducting qualitative interview research with people with expressive language difficulties. Qualitative Health Research.

[8] Gerritsen DL (2007). Measurement of overall quality of life in nursing homes through self-report: the role of cognitive impairment. Quality of Life Research.

[9] Dalemans R (2010). Psychometric properties of the Community Integration Questionnaire adjusted for people with aphasia. Archives Physical and Medical Rehabilitation.

[10] McHorney CA (2000). The SWAL-QOL outcomes tool for oropharyngeal dysphagia in adults: I. Conceptual foundation and item development. Dysphagia.

[11] Khaldoun E, Woisard V, Verin E (2009). Validation in French of the SWAL-QOL scale in patients with oropharyngeal dysphagia. Gastroenterologie Clinique et Biologique.

[12] Rinkel RN (2009). The psychometric and clinical validity of the SWAL-QOL questionnaire in evaluating swallowing problems experienced by patients with oral and oropharyngeal cancer. Oral Oncology.

[13] Lam PM, Lai CKY (2011). The validation of the Chinese version of the Swallow Quality of Life Questionnaire (SWAL-QOL) using exploratory and confirmatory factor analysis. Dysphagia.

[14] McHorney CA (2002). The SWAL-QOL and SWAL-CARE outcomes for oropharyngeal dysphagiain adults: III. Documentation of reliability and validity. Dysphagia.

[15] Bogaardt HCA (2009). Cross-cultural adaptation and validation of the Dutch version of SWAL-QOL. Dysphagia.

[16] Fleiss JL (1981). Statistical methods for rates and proportions.

[17] Mokkink LB (2010). The COSMIN checklist for assessing the methodological quality of studies on measurement properties of health status measurements: an international Delphi study. Qualitative Life Research.

[18] Buck D (2004). Development and validation of NEWSQol, the Newcastle stroke -specific quality of life measure. Cerebrovascular Diseases.

